# New York Institute of Stomatology

**Published:** 1895-07

**Authors:** 

**Affiliations:** pro tem., New York Institute of Stomatology


### NEW YORK INSTITUTE OF STOMATOLOGY.

A MEETING of the New York Institute of Stomatology was held at the residence of Dr. C. A. Woodward, No. 49 West Forty-sixth Street, Friday evening, April 19, 1895, Dr. J. Stedman Converse presiding.

*The Chairman.*—Gentlemen, we will omit Communications on Theory and Practice this evening and listen to Dr. Allan, who will make a statement for the members of the Institute.

*Dr. Geo. S. Allan.*—It has seemed wise to the gentlemen interested in this movement to state briefly their reasons for starting a new society, why they think it has a right to live, and why they believe that it will grow and prosper.

In the name chosen and in the plan of organization we would recognize, first, the enlarged position which the profession occupies to-day, and, second, our purpose to make the educational idea the most prominent one. We purpose to occupy the dual position of pupil and teacher, and to make our organic law broad and deep enough to compass all the possibilities of the future. The word "stomatology" better represents the status of the profession to-day than does the more familiar one "dentistry," and it defines more exactly the true position of dentistry as a specialty of medicine.

As all sciences are closely related and interdependent, and no one can grow as a whole or in its units (its members) standing alone, so this institute will seek to bring the science of dentistry into closer relations with all other sciences, kindred ones especially, by opening its doors to all who can bring food or encouragement by adding to our knowledge or enlarging our powers of thought.

As an institute we hope to obtain all the modern facilities for acquiring and imparting knowledge respecting our science. This naturally means the ownership of a library and museum, a place for work and study, and it especially means favoring a college based on the fact that as dentistry is a specialty of medicine so the graduates of the dental school must be in truth as well as name "doctors of medicine."

If a society would be strong it must in all things be subordinated to the principle and the purpose upon which it has been founded. Great care, moreover, must be taken at the outset to eliminate all sources of weakness and discord, and to encourage whatever will give strength and power.

As our sole ambition is centred in the welfare and growth of the profession, individual claims must be based not upon personal grounds, but upon the amount of good done for the common cause. Society politics and log-rolling for position will be made impossible: too often have we witnessed their disintegrating and baneful effects.

This movement is in no way a hostile one; not only do we not antagonize any existing society, but we, on the contrary, recognize and appreciate all their good work, both of past and present. The reading and discussing of papers, and the holding of clinics is essential and valuable, and we have no expectation of dropping or of slighting them. We do not, however, purpose to make these either our sole or our most important work. We hope to attain our ends to a great extent by other means.

Actuated as we are by common thoughts and aims, we think it better to start anew than to attempt to force new ideas into prominence in either of the older organizations. In this way we hope to avoid the objections and antagonism of those who simply do not see things as we see them, and who would rather, therefore, walk in their accustomed way than follow in our footsteps. The Institute as far as possible will be composed of workers, and all those who will work for the common cause will be gladly welcomed. But they must be in sympathy with us and wear the same harness.

*The Chairman.*—Gentlemen, I have the pleasure of introducing Professor Henry F. Osborn, of the Department of Biology, Columbia College, who will deliver an address upon the subject, "The History of the Cusps of the Human Molar Teeth."

(For Professor Osborn's paper, see page 389.)

DISCUSSION.

*The Chairman.*—Gentlemen, Professor Osborn's scientific paper is before you for discussion, and I hope many will participate. We have with us this evening Professor Peirce, of Philadelphia; we would like to hear from him on this subject.

*Dr. C. N. Peirce.*—Mr. Chairman, before responding to your invitation it will give me great pleasure to introduce to you Dr. J. L. Wortman, of your own city, a gentleman to whom the dental profession is indebted for one of the most complete papers on the comparative anatomy of the teeth that has ever been published, and the only one of any importance found in a publication of this country.

*Dr. J. L. Wortman.*—Mr. President and gentlemen, I certainly do not know what I can add to the remarks that have been already made by Professor Osborn, unless I may be permitted to say something more in regard to the general bearing of this view of the origin of multicuspid or complex teeth of the mammalia from the tritubercular form. When I say the tritubercular form, I do not mean to state that this is by any means the lowest or simplest form to which the structure of mammalian teeth can be traced, but it rather represents a stage in tooth-evolution which was at least exceedingly common to the mammals in early geological times, and to which the evidence of palæontology leads us apparently with irresistible force. If, therefore, multicuspid teeth have originated from tricuspid forms, it is but fair to presume that these in turn have gradually arisen from still simpler types, and we consequently go back to the unicuspid form as the common ancestor of all. The evidence of the evolution of the tricuspid form from the unicuspid, however, is not so strong nor conclusive as it is of the multicuspid from the tricuspid or tritubercular.

I am aware of the fact that there are some objections to this view, and I often think that they are very serious objections. One of these objections is that among the earliest mammals of which we have any knowledge we meet with forms (Plagiaulacidæ) whose teeth are almost, if not quite, as complex in structure, so far as the number of cusps is concerned, as the most specialized forms living to day. The whole group is, in fact, known as the Multituberculata, which gives you at once a clue to the complexity of their molars.

Now, if we attempt to explain this early, complex tooth upon the hypothesis that it has originated from a single cone, allowing the same rate of evolution as we do for the later forms of equal complexity, we will be compelled to place the origin of the mammalia so far back in time as to do violence to the facts. We must either suppose that its evolution was exceedingly rapid or that it was derived from a form which had already become complex in its reptilian ancestors. This latter alternative involves a polyphyletic origin of the mammalian group, which I think is not wholly unwarranted by the evidence already in our possession. We would have then one group of mammals springing from reptiles with comparatively simple, more or less conical teeth, and another coming from reptilian forms with complex teeth.

With reference to the complexity of the teeth among the lower forms of the vertebrata, I will say that it is by no means an unusual occurrence; I have only to call to your attention such examples as Notidanus and Cestracion among the sharks, the teeth of the Chimæroids and Rays among an allied group, the teeth of the "lung fishes" (Dipnoans), and lastly the complex teeth of many of the Theromorph reptiles, which might easily have given origin to the Multituberculate mammals. It is, indeed, highly improbable that such structures as the complex teeth of these lower forms I have just mentioned, with the possible exception of those of the Theromorph reptiles, have anything like the same history as the multicuspid teeth of the mammal. In one the complexity was probably impressed upon it while it was yet intimately connected with the skin, while in the other it was only after it had developed a fixed connection with the bones taking part in the boundary of the mouth cavity; and this primitive structure we have reason to believe was a simple cone.

Taking this as our starting-point, therefore, in the developmental history of the mammalian tooth, let us examine briefly the two hypotheses to which Professor Osborn has just called your attention,—viz., cusp addition by means of a union of two or more of these primitive cones; the theory of concrescence, or cusp addition by means of outgrowths from different parts of the primitive cone, the implied hypothesis of the tritubercular theory. In reviewing the evidence in favor of each of these hypotheses, I do not know as I can do better than recall a remark that was once made to me by a very distinguished student of biology many years ago, before I understood much of the doctrine of evolution. I asked him, "What do you consider to be the strongest evidence in favor of evolution?" His reply was this: "If we look at the evidence from the stand-point from which Mr. Darwin has so elaborately reviewed it, a study of the living forms, the truth of the hypothesis is rendered possible. If we look at the evidence from the stand-point of embryology, a study of the becoming of the individual, then the truth of the hypothesis is rendered highly probable; but if we examine the evidence from the stand-point of palæontology, a study of the remains of individuals that have actually existed in past time, then the truth of the hypothesis is rendered absolutely certain." I quote this remark simply to show you the relative value of the evidence drawn from these various sources in determining the evolution of any organ or set of organs whose development we seek to explain.

Fortunately, the geological record has now approached that stage of completion which enables us to construct at least a part of the pylogenetic history of a number of important groups of mammals with an unusual degree of certainty, and from them we can gather some very direct and positive evidence regarding the evolution of the teeth. I will mention a few examples which are doubtless well known to you: these are the horses, rhinoceroses, tapirs, and the Titanotheres, although numerous others could be given. In them we note that in the earliest forms the premolars are simple, and that as evolution advanced they gradually assumed the complexity of the molars. These new additions, which were made to these originally simple, more or less conical, teeth, began as minute cuspules, ridges, etc., and we can actually see in a succession of species at what point and in what manner the additions were made. They were not developed by the union of separate and distinct tooth-germs, but were made by outgrowths to the single cone already in existence. The evidence of this is so powerful, so positive, and so conclusive that it seems to me, so far as these teeth are concerned, at least, there is no room for argument. Now, this is not only true of the evolution of the premolars in the lines I have just mentioned, but it is also true of the premolars of all forms whose tendency was in the direction of complexity. There is yet more: in some forms whose history we can trace with considerable certainty far back into Eocene time, we find not only simple premolars, but the true molars are reduced to the tritubercular pattern. This is seen in the early even-toed ungulates as well as the lemurs. With this I will say our positive and direct knowledge ends, for the reason that beyond this point we are unable to trace the lines of descent with that same accuracy and certainty which we can in the later geological epochs. While it is true that we know many species from the lowermost Eocene, which in all probability were the ancestors of the later types, yet we cannot demonstrate this beyond question until we know more of their entire skeletal structure. It is a significant fact, however, that, barring the Multituberculates, all these forms, with two exceptions, have the molars organized upon the tritubercular pattern.

When we go beyond the Eocene into the Cretaceous, Jurassic, and Triassic formations, we meet with mammalian remains representing a number of different species, but they are known only from the most fragmentary specimens. It is impossible to trace phyletic lines among them, in consequence of which any deductions which we draw from a study of their teeth are more or less speculative.

Turning now to the other hypothesis, or that of concrescence, we find that the evidence is almost entirely of a speculative or a negative character. The only positive evidence which has yet been advanced in its favor is drawn from embryology, and this we are led to mistrust, if not reject altogether, as either misleading or useless. The most earnest advocate of this view, Dr. Carl Röse, has discovered that the various cusps of a mammalian molar begin to calcify at so many separate points, just as you have separate centres of ossification in a compound bone. He concludes from this that each one of these separate points of calcification represents an originally separate and distinct tooth-germ. This view he believes to be further strengthened by the fact that the dates at which calcification of these various cusps begins are slightly different.

Let us examine this question of the development of a single tooth a little more in detail and see, if possible, what bearing it has upon this theory. You are all so familiar with this process, I take it, that I need mention only the leading facts. You know, for example, how the enamel organ is formed, and you know also how the dentinal papilla is formed, and it strikes me as an exceedingly curious circumstance that if embryology is going to give us any light upon this question it should be so singularly silent at the very point where we would most naturally expect to find the evidence if this theory of concrescence is true. With all that has been written on the subject of the development of the teeth, including the researches of Röse, Kükenthal, Leche, Fleischmann, and others, the dentinal papilla has never been observed to consist of more than a single body from the very moment it begins to grow. If it is a compound body, representing the coalescence of a number of separate dentine germs, why, may I ask, do we not get some evidence of this union when it is first formed? What is here said of the dental papilla applies with equal force to the enamel organ. It, too, always arises as a single and not a multiple diverticulum of the parent fold. The fact that calcification begins at several points in either the enamel germ or the dental papilla or both, I do not regard as any proof whatever that either is a compound structure, especially when we consider that both arise as single organs.

This process of calcification, I am led to believe, belongs in the same category as the ossification of a bone. Take the humerus, for example, in the human body, and we find no less than seven centres where the lime-salts commence to be deposited. In the shaft, ossification begins at about the fifth week of fœtal life, in the head at from the first to the second year, in the internal condyle at about the fifth year, in the external condyle at the thirteenth or fourteenth year, and so on. Again, if we study the human femur we will find that there are five centres of ossification, which appear all the way from the fifth week to the fourth year of the life of the individual. Now, who has ever ventured to assert from these facts that either of these bones is a compound bone, made up in the one case of five and in the other of seven elements, and that the time they commence to ossify has anything whatever to do with the order in which they have been added? What ancestor of the human family had such a remarkable femur or humerus as to be composed of five or seven separate bones? By this I do not mean to assert that the centres from which bones commence to ossify is without its special value in the determination of the homologies of parts in many cases, but at the same time there is much evidence of this character that is worthless and meaningless, and I am inclined to regard this evidence of calcification of the tooth-germ as coming entirely within this domain. Truly, gentlemen, I cannot see any evidence for such a view.

*Dr. C. N. Peirce.*—Mr. Chairman, I want to express my gratification in being with you this evening and the very great pleasure I have had in listening to the addresses of Professor Osborn and Dr. Wortman. I have been well compensated for my little trip here from Philadelphia.

Accepting as I do the theory of evolution, I can agree most emphatically with the theory that has been suggested this evening of the derivation of the more complex teeth from the simple.

If you will pardon me, I want to make some allusion to an occasional condition found at the present day, believing it has some bearing upon the subject under discussion. It is not uncommon to hear certain aberrations in development spoken of as a reversion to an ancestral type, and one of these mal-developments is the congenital union of the crowns of both the deciduous and permanent anterior teeth, the incisors only being involved in it. The posterior teeth,—molars and bicuspids,—if ever, are so rarely united in their crowns (congenital) that they have escaped recognition in this form of union, though union of their roots, an acquired union, is frequently observed. That this abnormality should be confined to the anterior teeth I have thought rather remarkable, unless it can be attributed to the crowding of these germs, while the posterior teeth are in their development less under this influence.

Some years ago I published a little paper, in which I stated that while the anterior teeth were liable to this abnormality, it was never recognized in the posterior; some few weeks after the publication I received from a gentleman in Virginia a tooth; he said he sent it because it represented the union of two bicuspids which had been filled with gold, and it proved my statement to be incorrect. I wrote him at once that I had received his specimen, and that it was well filled, but that I judged his patient grunted when it was extracted, though of one of the first families. The union of bicuspids I have never seen. The molars of the pig do very much resemble the union of two human bicuspids.

I certainly can add nothing to what has been said regarding the formation of the teeth ; but the question comes constantly in my mind as to what has been the factor that has induced this modification. Has it been the food habit, and the movements of the lower jaw upon the upper? I certainly have so surmised. Some of you may recall an article I published in the *Dental Cosmos* a few years since; it was a synopsis of a paper by the late Dr. John A. Ryder on this subject. He prepared quite an extensive essay, well illustrated, showing the influence of the lower teeth upon the upper. It was his belief that the movements of the lower jaw and the impact of the teeth upon each other had modified the physical character of the teeth. The paper was entitled "The Mechanical Genesis of Tooth Forms," and was certainly a very interesting one, showing that the movement of the inferior upon the superior jaw certainly had had a marked influence in the arrangement of the cusps of the molars. When we see the change from a simple cone to the complex tooth, we ask, very naturally, What has been the factor that has induced it? Has it been due to a modification of food, and with this a modification in the movements of the lower jaw upon the upper?

It has always been a great satisfaction to me to feel that the *Lamarckian* theory of use and disuse was a correct interpretation of a great factor in the organization of structures. And believing firmly in this, I have looked upon the food habit as the factor in the development or evolution of the complex tooth from the simple cone, and also recognized its influence in the number and arrangement of the cusps. This theory has been very useful to me in my practice, and as I am talking mostly to dentists, I may touch upon the practical side of this question. I have recognized the fact that where patients use their teeth for the trituration of hard substances, they were in much better condition than where this use of the teeth was much neglected and the food simply bolted with moisture and without mastication. It has been my habit to urge patients that the use of their teeth was essential to their preservation, and to impress upon parents the fact that the proper development, as well as the preservation, of their children's teeth required that they should take their food dry, with plenty of time for its thorough mastication. I have certainly recognized a very great change for the better in the condition of the mouths of patients, so far as preservation of the teeth is concerned, since I have insisted that the natural use of the teeth should be observed in the proper mastication of the food.

I would like to ask Professor Osborn to give us what he deems the important factor or factors in the modification of simple cones into complex teeth; whether he does not think that the fulfilment of the Lamarckian theory has been instrumental in making the changes. It has always been a theory of mine that such was the case, and that the shape of the teeth was largely due to their use, believing that use and disuse were very important factors in the status of the teeth.

*Dr. Osborn.*—Mr. Chairman, I am very glad that Dr. Peirce has asked that question, because I think it is undoubtedly the case, as he has observed in his practice, that the teeth are modified by use. A comparison of the teeth of savages with those of Europeans will support that conclusion. Savages usually chew hard food, or, like the Australians, use their teeth in place of tools; and their teeth are found in fine condition, often completely worn down. The teeth of the Australians are often found to be worn down almost to the gums, with little decay, while the teeth of Europeans and Americans are little worn and much decayed in comparison, as you know. It is undoubtedly the case, as Dr. Peirce has suggested, and as the late Professor Ryder worked out beautifully, that the points of maximum use in the teeth are points in which the new cusps appear. When you look at the posterior lingual in the upper molars, for instance, you see that this cusp corresponds to the point at which the primitive anterior lingual would come in contact. So that use of the teeth seems to have brought about an increased amount of wear. In some of the monkeys' teeth which I have observed, in the later Eocene period, the upper teeth have somewhat of a triangular form.

You remember what I said in regard to the loss of the primitive anterior lingual in the lower molars; that has been one of the greatest difficulties we have met with in the application of this "*use*" hypothesis. When we go back to the complete triangle here in the lower jaw, the primitive anterior lingual will evidently wear against the posterior base of the anterior palatal in the upper jaw, and the point will be worn down or bear the maximum wear. Now, here comes the difficulty: as the new posterior palatal in the upper molar develops, the primitive anterior lingual in the lower molar disappears; so that we have one cusp constantly getting larger and the other rubbing against it but constantly getting smaller, until finally the latter disappears entirely from the human molar and the former becomes very large. Now, this is a mechanical paradox, because it is obvious that the action and reaction between the lower cusp and the upper must have been equal. There are a number of facts of this kind which make it very difficult to apply this mechanical theory to the development of tooth forms. Looking at certain facts, you find that everything points towards the mechanical theory of cusp development, but when you go through the whole scale of the different types and analyze each case you meet many difficulties. My whole inclination is towards the mechanical theory; it seems a most beautiful theory, and as an evolutionist who believes that things are worked out according to certain laws and not wholly by chance, so as to provide for the survival of the fittest, I find plenty of fitness in this theory. You find that use of parts of the body improves them, and if we apply that use of parts and the consequent improvement through heredity we have an explanation of the origin of a great many useful and beautiful adaptations in nature. We notice that our big toe is very much larger than it was and the little toe is disappearing, so that ultimately we are becoming one-toed animals. That is because we have learned to walk on the inner side of the foot, the weight of the body resting on the great toe, so that the main resistance comes against the great toe; that is a mechanical explanation of the fact that in all civilized races the great toe has become very much larger than the little one.

It seems to me, however, that whenever we apply a theory to every case, we do meet with some very great difficulties.

*Dr. Peirce.*—In the development of the cusp of the first molar especially we have the anterior buccal cusp calcified first, then comes the posterior buccal, and next the anterior palatal in the upper molar. The anterior buccal cusp is the first, and the three are invariably calcified before the fourth distal palatine.

*Dr. Osborn.*—In the upper molar?

*Dr. Peirce.*—It is a significant fact that we oftentimes find small spurs on the molar teeth, anterior spurs, more frequently on the antero-palatal surface than on the buccal; so we have what you might call five- or six-cusp teeth at times.

Then in the development of the teeth where one tooth is impacted between two others and is making its way gradually to its place, that tooth is modified in shape by the impact of the other teeth; there is not an imaginary but a real compression of the crown of the tooth, so that it is fitted compactly in between two other teeth.

*Dr. Osborn.*—In regard to that anterior spur that Dr. Peirce has spoken of, it is a very interesting element in the comparative anatomy of the teeth. It occurs at the anterior base of the anterior upper lingual, does it not?

*Dr. Peirce.*—Yes.

*Dr. Osborn.*—That spur is found in a large number of the lower forms of mammals. In some forms it is a persistent feature of the teeth, and occurs as a family characteristic, but in some species it seems to occur occasionally only as a spur. I think it indicates that there is a latent tendency to develop a cusp at that point. In a large number of types of animals you will find that spur strongly developed.

You have no idea, gentlemen, how important and valuable are the observations which you make in your practice or how interesting they are to the comparative anatomist. It is the one great drawback of modern specialization that the specialist has gone so far in a particular direction away from the general practitioner that men are not brought together sufficiently to compare notes; and I think one of the great services that a society like this can render is by the publication of observations, so that others may have the opportunity of seeing the bearing of these points, such as the development of the wisdom-teeth, the development of the anterior and posterior teeth, the order of calcification of cusps, and the occasional appearance of a third series of teeth in the jaw. The evidence of a third series of teeth was first noticed, I believe, by a practising dentist, and now it has been positively shown that some of the mammalia exhibit vestiges of a third set of teeth, for underneath the permanent bicuspids are the rudiments of a third series that sometimes come to the surface. If scattered observations could only be collected, so that you gentlemen of the dental profession could take advantage of our observations and we could take advantage of the observations that you make, then dental science would progress very much more rapidly than it does.

*The Chairman.*—We would be pleased to have Dr. Stebbins address us upon this subject.

*Dr. R. O. Stebbins.*—Mr. Chairman, calling attention particularly to the condition of these teeth [referring to an Eskimo skull and denture], it will be noticed that the incisor teeth, as a rule, are large and very blunt, which accounts for the statement made in several reports that the natives had double teeth all around. It was my pleasure to visit the far North last summer and ascertain the true character of the Eskimo teeth, both of the original race and the present tribe of natives known as Danish Eskimos or Greenlanders.

Originally the natives lived entirely on fish and flesh, having no vegetable or bread-stuff of any kind. They did not cook their food, using their incisor teeth to cut off bits of meat or fish, and swallowing it immediately. Their molar teeth did not come into play as masticators, which would account for the wearing away of the front teeth; also, their manner of dressing bird and other skins, which is accomplished by constantly chewing the skin to make it pliable, tearing off bits of flesh, and sucking the oil and grease out.

Skins thus prepared are beautifully cured at the expense of wearing the teeth away. The statement that the Eskimos live on blubber is partly true: through the long Arctic nights they eat blubber and drink oil to keep warm, while through the corresponding long day they subsist on raw fish and flesh.

On one occasion we caught some trout, and were asked by a little fellow if he could have one to eat; he grasped it by the head, and, biting off a piece of the fish, which was wriggling in his hand, gulped it down, and then another piece, till he had swallowed the entire fish.

Since the Danish government has introduced vegetables and bread-stuffs, together with the cook-stove, the natives cook more or less of their food, which they have to chew, as they cannot swallow bread without mixing saliva with it to assist digestion, which is not the case with a meat and fish diet exclusively. I took twenty-eight impressions of teeth, from childhood to old age, making plaster casts of both upper and lower jaws. I also visited some of the old graveyards, which are found on the mountain-sides, to obtain skulls. There being no earth, and consequently no trees of any kind, the bodies are covered with moss and stones. The Greenlanders or present tribe of Danish Eskimos bury their dead near the water's edge.

In many cases the first- or sixth-year molar teeth were decayed, while not a sign of decay was found in other teeth in the same mouth. Since the present natives chew their cooked food, one notices that the upper incisor teeth project over the lower teeth, which was rarely the case in the older tribes.

*Dr. Allan.*—Mr. President, I am very glad that on our opening night our proposed work has been brought to the front in such an admirable shape, and I am sure we will pursue it harmoniously.

I had some manuscript with me, but the lateness of the hour prevents me from making use of it now.

Professor Osborn has anticipated some of the questions that I wanted to ask, but I would like to ask Dr. Wortman, who referred to Dr. Carl Röse's ideas about the fusing together of separate teeth to form the multicusp tooth that we have in the human molars, whether the embryonic conditions that Dr. Carl Röse gives such great importance to have been fully answered by Professor Osborn or not. I am not in a position to answer. One thing that Dr. Wortman said struck me as a little peculiar,—that the development of the cusps proceeded, as you might say, harmoniously. If I had been asked that question I should have said no, but I am not certain about it. My impression is that histological observations would confirm Dr. Carl Röse's idea, that they do not proceed harmoniously, or, rather, do not start precisely at the same time and proceed at the same rate in the calcification. One thing is certain, whether the cusps commence calcification at the same moment or not, it is true that they do not proceed with the same rapidity; calcification proceeds faster in some cusps than it does in others. Where the cusps come together and coalesce we have fissures that are the bane of the dentist's life: there is a more or less open crack, which invites decay and makes trouble. Now, that would in some way seem to suggest the thought that possibly single cusps, in their fusion together to form a multicusp tooth has some foundation from a histological stand-point. I cannot, of course, give any opinion upon it, but I would like to know what bearing it has on this theory of development.

*Dr. Wortman.*—I will say that I feel the importance of the evidence that embryology furnishes us, in many cases, when we are going to trace the evolution of this or that part of an animal structure; in a majority of instances embryology will give us a clew. I cannot say that I am myself an expert on the subject of development of the teeth, although I have studied the subject with some care. In searching the literature on this question at the time I was engaged on my work I failed to find any evidence that there was a difference in the time that the different tooth-cusps appear. Taking into consideration the statements of Tomes, who was the last writer on the subject at the time of my publication (1886), I was led to reject the idea that embryology gives us any clew whatever to the history of cusp addition. And when we come to review the subject in the light of these more recent investigations, I do not see that the evidence derived from this source has been materially strengthened.

*Dr. J. Morgan Howe.*—Mr. Chairman, I wish to express the great interest and pleasure I have had in this lecture of Professor Osborn's, and in the discussion of it, both by Dr. Wortman and Professor Peirce; and I move a vote of thanks to each one of these gentlemen for the trouble and care that they have taken in presenting this subject to us this evening.

Dr. Howe's motion was carried.

Adjourned.

S. E. Davenport, D.D.S., M.D.S.,
*Editor pro tem., New York Institute of Stomatology.*

